# DPP6 Loss Impacts Hippocampal Synaptic Development and Induces Behavioral Impairments in Recognition, Learning and Memory

**DOI:** 10.3389/fncel.2018.00084

**Published:** 2018-03-29

**Authors:** Lin Lin, Jonathan G. Murphy, Rose-Marie Karlsson, Ronald S. Petralia, Jakob J. Gutzmann, Daniel Abebe, Ya-Xian Wang, Heather A. Cameron, Dax A. Hoffman

**Affiliations:** ^1^Molecular Neurophysiology and Biophysics Section, Program in Developmental Neuroscience, Eunice Kennedy Shriver National Institute of Child Health and Human Development, Bethesda, MD, United States; ^2^Section on Neuroplasticity, National Institute of Mental Health, Bethesda, MD, United States; ^3^Advanced Imaging Core, National Institute on Deafness and Other Communication Disorders, National Institutes of Health, Bethesda, MD, United States

**Keywords:** DPP6, neurodevelopment, learning and memory, autism spectrum disorder

## Abstract

DPP6 is well known as an auxiliary subunit of Kv4-containing, A-type K^+^ channels which regulate dendritic excitability in hippocampal CA1 pyramidal neurons. We have recently reported, however, a novel role for DPP6 in regulating dendritic filopodia formation and stability, affecting synaptic development and function. These results are notable considering recent clinical findings associating DPP6 with neurodevelopmental and intellectual disorders. Here we assessed the behavioral consequences of DPP6 loss. We found that DPP6 knockout (DPP6-KO) mice are impaired in hippocampus-dependent learning and memory. Results from the Morris water maze and T-maze tasks showed that DPP6-KO mice exhibit slower learning and reduced memory performance. DPP6 mouse brain weight is reduced throughout development compared with WT, and *in vitro* imaging results indicated that DPP6 loss affects synaptic structure and motility. Taken together, these results show impaired synaptic development along with spatial learning and memory deficiencies in DPP6-KO mice.

## Introduction

DPP6 is well known as an auxiliary subunit of Kv4-containing A-type K^+^ channels (Nadal et al., 2003) that regulate the excitability and plasticity of neurons and other excitable cells (Hoffman et al., 1997; Spruston and Johnston, 2008). DPP6 co-expression enhances Kv4 channel surface expression, increases conductance and accelerates channel activation, inactivation and recovery from inactivation (Nadal et al., 2001; Kaulin et al., 2009). DPP6 is a type II transmembrane protein with about 90% of the protein located in the extracellular C-terminus and only a short intracellular sequence following a single transmembrane domain (Strop et al., 2004). We have reported previously, using a heterologous expression system, that the intracellular N-terminal and transmembrane regions of DPP6 associate with Kv4.2 and accelerate channel kinetics. However, the extracellular domain is required for DPP6 subcellular trafficking and promotes Kv4.2 surface expression (Lin et al., 2014). Our previous study of DPP6 knockout (DPP6-KO) mice showed that decreased A-type K^+^ current expression in CA1 hippocampal pyramidal neuron distal dendrites leads to hyperexcitable dendrites, enhanced dendritic action potential back-propagation, increased calcium electrogenesis, and a lowered threshold for the induction of long-term synaptic potentiation (Sun et al., 2011).

Clinically, the DPP6 gene has been associated with numerous developmental and intellectual disorders and neuropsychiatric pathologies but perhaps most consistently with neurodevelopmental disorders (Liao et al., 2013; Egger et al., 2014; Bock et al., 2016). Autism spectrum disorder (ASD) is a neurodevelopmental disorder generally characterized by impairments in communication, social interaction and repetitive patterns of behavior. These complex behaviors depend on the ability to process and bind together multiple streams of sensory and motor information along with stored memory representations within the context in which experiences occur. Such binding has been proposed to depend on the hippocampus (Olsen et al., 2012). Accordingly, the hippocampus has been recently found to be necessary for social memory in mice (Hitti and Siegelbaum, 2014). Although altered hippocampal function has been suspected to play a role in the etiology of ASD, little direct patient information exists given the complexity of behaviors involved and lack of hippocampal specific tests in humans, although anatomically the hippocampus was found to be enlarged in ASD patients of all ages (Schumann et al., 2004).

DPP6 regulates hippocampal synaptic development and function and has been implicated in neurodevelopmental disorders that impact learning and memory. However, the behavioral effects of DPP6 deletion has yet to be determined. Here we report that DPP6-KO mice show deficits in various learning and memory tasks, including the Morris water maze, rewarded alternation in T-maze task, contextual fear conditioning task and object recognition. Ultrastructural examination by electron microscopy revealed fewer synapses and smaller spines in the hippocampal CA1 region of DPP6-KO mice. Time-lapse imaging of dendritic spines in hippocampal neurons cultured from DPP6-KO mice show increased spine motility, supporting the view that DPP6 plays a structural role in synapses in addition to regulation of A-type K^+^ channel function. These findings indicate that DPP6 is critically involved in synapse formation and the regulation of hippocampus-dependent learning and memory.

## Materials and Methods

### Animals

DPP6-KO and WT littermate mice were weaned at 3 weeks of age, genotyped via PCR, and housed 3–4 per cage with mixed genotype siblings. Mice were housed under a 12-h light/dark cycle with the lights off at 18:00. Behavioral experiments were performed in an adjacent dedicated procedure room. Mice were habituated to the test room for at least 1 h prior to start of the behavioral tasks. Couple sets of male mice aged 8 weeks (~10 mice for WT and DPP6-KO each set) were used for the behavioral tasks. Those that involved multiple tests began with the least stressful tests (e.g., open field, home cage) before the more stressful tests (Water-maze and T-maze). All animal procedures were performed in accordance with guidelines approved by the National Institute of Child Health and Human Development Animal Care and Use Committee and in accordance with NIH guidelines.

### Morris Water Maze Task

The Morris water maze task was performed to evaluate hippocampus-dependent spatial navigation learning and memory (Morris, 1981, 1984; Vorhees and Williams, 2006). The water maze consisted of a 120 cm circular pool (depth 50 cm), filled 40 cm deep with room temperature 22°C water made opaque with nontoxic white paint and containing a 10 cm diameter platform. External high contrast cues were placed about the room and on the interior of the pool above the water surface to aid spatial navigation. Trials were video recorded and scored by ANY-maze software (ANY-maze, Wood Dale, IL, USA) for measures including latency to find the hidden platform, total distance traveled and swim speed. On Day 1 mice were trained in the visible platform version of the Morris water maze task in which the platform is 1 cm above the water surface with a red flag placed on the platform to increase its visibility. On Day 2 through Day 5 mice were trained for the Hidden Platform version where the flag was removed from the platform and additional water was added to the pool to submerge the platform to 1 cm below the surface. Each mouse was placed into the water maze, facing the wall, in one of four possible quadrant positions, which pseudo-randomly differed by training day. Mice were given 60 s to find the hidden platform and given a ~5 s platform rest interval. If a mouse was unable to find the platform in the allocated time, it was gently guided to the platform and allowed to rest for ~10 s. Mice were then put back to the cage for 15 s after each trail. Mice were given a total of 20 training trials. On Day 6, the platform was removed and mice underwent a 60 s probe trial to determine the amount of time spent exploring the target quadrant and the number of times the animal crossed the previous platform location.

### Rewarded Alternation in T-Maze Task

Rewarded alternation on the T-maze to test spatial working memory (non-matching to place) was carried out using a clear Plexiglas T-maze, placed on a white table. The maze consisted of a start arm (31 cm) and two identical goal arms (30 cm), surrounded by a 12.5 cm high wall. Prior to testing, mice were food restricted to 85%–90% of their free-feeding weight and habituated to the maze, and to the 50% sweetened condensed milk reward (diluted with water) over several days before rewarded spatial alternation testing began. Each trial consisted of a sample run and a choice run. At the start of each trial, 0.1 ml of the diluted sweetened condensed milk reward was placed in the food wells at the end of each goal arm. On sample runs, the mouse was forced either left or right by blocking access to one goal arm with a clear Plexiglas door according to a pseudo-random sequence. During the choice run, mice were given a free choice of either goal arm. A trial was scored as “correct” if the mouse entered the previously unvisited arm. The delay between sample run and choice run was 10–15 s. Mice were tested with an inter-trial interval of ~10 min and received five daily trials for a period of 5 days.

### Contextual Fear Conditioning Task

Contextual fear conditioning was conducted in a clear-walled chamber with internal dimensions of 30 × 30 × 24 cm (ActiMetrics, Wilmette, IL, USA) with a metal-rod floor. After a 120 s acclimation period, there were three pairings (60–120 s inter-pairing interval) of the acoustic conditioning stimulus (CS; 20 s, 2 kHz, 75 dB) and the unconditioned stimulus (US; 1 s, 0.5 mA scrambled foot shock), in which the US was presented during the last 1 s of the CS. The session ended 120 s after the final CS-US pairing. The chambers were cleaned with 70% EtOH after each session. Twenty-four hours after this test session, mice were placed into the chamber for 8 min to assess context conditioning. Freezing activity was measured using Freeze Frame 4 software (ActiMetrics, Wilmette, IL, USA).

### Hot Plate Task

The Hot Plate apparatus (IITC Inc., Woodland Hills, CA, USA) was set to a temperature of 55°C. The mouse was introduced onto the surface of the hot plate with an open-ended cylinder. A remote foot-switch pad was used to control the start/stop functions. The latency to show a nociceptive response with hind paw lick was recorded. The mouse was immediately removed once this response is observed. A 30 s cut-off time was assigned in this protocol.

### Novel Object Recognition and Object Spatial Recognition Task

Recognition memory was tested in the same arena as the novelty-induced locomotor activity (Fernandez et al., 2007). In the novel object recognition test, during training, mice were placed into the experimental arena with two identical plastic objects (shaped like a butterfly, 8.5 × 7.5 × 2.5 cm) and allowed to explore for 10 min. Twenty-four hours later a novel object preference test (10 min) commenced where the mouse was placed in the arena and presented with two objects in the same position as at acquisition; one object was the same as used in the acquisition phase and the other was a novel plastic object (shaped like a cupcake, 7 × 7 × 7 cm). One week later mice were tested in the object spatial recognition task. In the acquisition phase the mouse was exposed to two similar plastic objects (shaped like a video camera, 7 × 7 × 4 cm) which were placed in the far corners of the arena. The mouse could explore both objects during a sample phase of 10 min. After a delay of 5 min the test phase began in which one of the objects was placed in the corner adjacent to the original position so that the two objects were diagonal from each other. Both objects in the test phase were equally familiar but one was in a new location. The amount of time that the animal spends exploring the novel object is used as a measure of memory as animals tend to spend more time examining unfamiliar objects. Exploratory behavior was defined by the ANY-maze software (ANY-maze, Wood Dale, IL, USA). A recognition index defined as the amount of time exploring the familiar object (TA) or the novel object (TB) divided by the total time spent exploring both objects and multiplied by 100 was used to measure recognition memory: (TA or TB/(TA + TB)) × 100.

### Open-Field Task

Novelty-induced locomotor activity was assessed in a novel open-field square arena (50 × 50 cm) constructed of white Plexiglas as previously described (Karlsson et al., 2008). The mouse was placed in the arena and left to explore freely for 60 min. Their activity was video recorded and distance traveled and time spent in different areas of the maze measured. Results were analyzed with “ANY-maze” software (ANY-maze, Wood Dale, IL, USA).

### Home Cage Task

Locomotor activity monitoring in a familiar environment was assessed over 6 days in individual home cages under normal vivarium conditioning via the photocell-based Photobeam Activity System version 2 (San Diego Instruments, San Diego, CA, USA).

### Spine Motility

The spine motility assay was adapted from a previously published protocol (Korkotian and Segal, 2001). Mouse hippocampal neurons were maintained 14–16 days in Neurobasal-A medium supplemented with B27 and Glutamax on PDL/Laminin coated Lab-Tek^®^ II 4-well chamber dishes. Twenty-four to 48 h prior to experiments, neurons were transfected using Lipofectamine 2000 to introduce GFP and/or mCherry expression plasmids. Culture medium was replaced with Tyrode’s buffer and time-lapse images were gathered using a Zeiss LSM 880 with Airyscan and equipped with a 63× 1.4 NA objective and a heated stage insert set to 34°C. Z-stacks were collected at 1 min intervals for 20 min. Prior to analysis, Z stacks were collapsed into maximum intensity projections to generate a 2D time series. Using ImageJ software, time series were drift corrected and analyzed with the stack difference command in the Multi Kymograph plugin. Analysis generated an image series representing the change in pixel intensity between frames resulting from spine movement. Images were then collapsed into a sum intensity projection representing the total movement during the imaging period. The resultant 2D image was thresholded to only the highest 2.0% of pixel intensities. This effectively excludes signals arising from the relatively immobile background fluorescence of dendrites and any *en passant* axons. Mean intensity of spine motion was normalized to spine number and then compared among dendritic fields of separate neurons from each condition.

### Mixed-Culture Assays for Analyzing Neuronal Synapse Formation

This assay was based on published protocols (Biederer and Scheiffele, 2007; Connor et al., 2016). HEK293 cells were transfected with pCAG-DPP6-GFP or control pCAG-GFP, and 24 h after transfection they were dissociated and seeded onto cultured hippocampal neurons. Mixed-cultures were maintained for 48 h before fixation. Cultures were stained for the pre-synaptic marker synapsin-555. Punctate synapsin intensity associated with HEK293 cells expressing GFP or DPP6-GFP was quantified using ImageJ software.

### Electron Microscopy

Electron micrographs used for analysis were originally collected for a previous study (Sun et al., 2011), and the preparation methods are based on a published protocol (Petralia et al., 2010). Briefly, rats were perfused with 4% paraformaldehyde plus 0.5% glutaraldehyde and sections were cut at 350 micrometers, cryoprotected and frozen in a Leica CPC (Vienna, Austria), and then embedded in Lowicryl HM-20 resin using a Leica AFS freeze-substitution instrument. After processing for immunogold (for the 2011 study), thin sections were stained with uranyl acetate and lead citrate, and were examined in a JEOL JEM-1010 transmission electron microscope. A random sample of micrographs was taken from the CA1 stratum radiatum of the hippocampus from a total of five each of WT and KO mice. Measurements were taken and analyzed in ImageJ. Area and perimeter of the spine profile in an image were measured by using the computer mouse to draw along the membrane of the spine head. Surface area and perimeter of the PSD profile in an image were measured by drawing along the edge of the density (thus, these are cross-sectional measurements of the PSD). To measure PSD length, a straight line was drawn along the long axis of the PSD. To measure PSD and cleft thickness, a line was drawn perpendicular to the postsynaptic membrane and, from the latter, either to the bottom edge of the PSD or to the presynaptic membrane, respectively. Statistical analysis was by Student’s *t*-test.

### Statistical Analysis

The *n* values, details of controls and comparisons used for statistical analyses are described for each experiment in the corresponding figure legend or within the “Results” section. Statistical analyses were performed using GraphPad Prism 6. We used one-way or two-way ANOVA (Holm-Šídák test *post hoc*) and Student’s *t*-test for all behavioral tasks. We used Student’s *t*-test for statistical analyses of spine mobility, mixed-culture assay, and synaptic structure by electron microscopy. All results are presented as the mean ± SEM.

## Results

### DPP6 Loss Leads to Learning and Memory Impairments

Following our previous studies showing that DPP6 impacts synaptic plasticity and synapse development and function in the hippocampus (Sun et al., 2011; Lin et al., 2013), we hypothesized that DPP6-KO mice would have impaired learning and memory behavior. To address this, we performed a battery of behavioral tests assaying both locomotor activity in novel and familiar environments and performance in learning and memory tests including novel object recognition, spatial object recognition, Morris water maze, and T-maze alteration.

### Morris Water Maze

**Figure 1:**
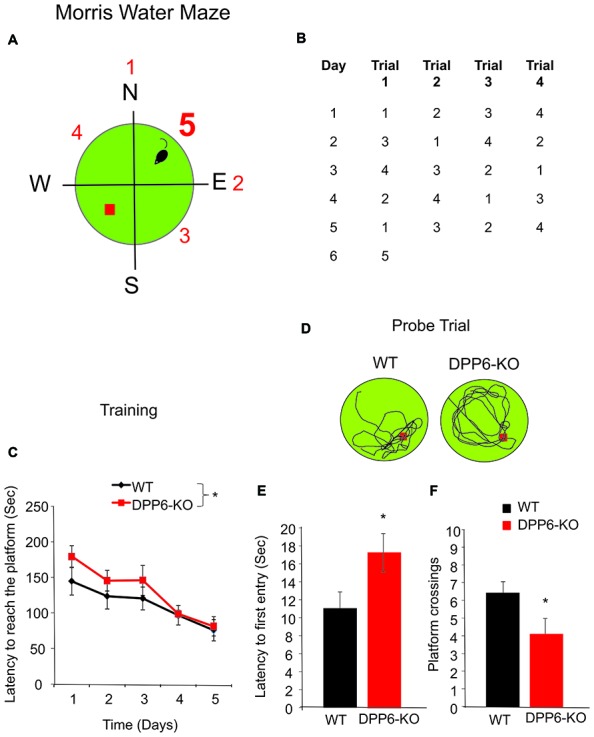
DPP6 mice show slower learning and memory impairments in the Morris water maze. **(A,B)** Schematic drawing showing the experimental design used to compare the performance of WT and DPP6 knockout (DPP6-KO) mice in the water maze. **(C)** Sum of latencies for four trials required to reach the platform in the water maze for WT (*n* = 10 mice) and DPP6-KO mice (*n* = 10 mice) over a training period of 5 days. **(D)** Representative swimming traces during the probe trial of the WT and DPP6-KO mice at day 6. **(E,F)** DPP6-KO mice are significantly slower to find the platform **(E)** and showed fewer platform crossings **(F)** compared to WT mice during the probe trail (*p < 0.05).

The Morris water maze test is often used to study hippocampus-dependent spatial learning and memory (Morris, 1981; Chen et al., 2006; Ip et al., 2011). In this test, both WT and littermate DPP6-KO mice were trained to learn and remember the same hidden platform location over 5 days by using spatial cues. Although the platform remained in the same position throughout training, the starting position changed on each trial to prevent the use of egocentric strategies to find the platform. We found DPP6-KO mice took significantly longer to locate the hidden platform compared to WT littermates in the training trials (Figure 1C), Two-way ANOVA, interaction between genotype and day (*F*_(4,90)_ = 11.65, *p* < 0.05). Importantly, the average swimming speed was not different between genotypes (WT = 22 ± 1 cm/s, *n* = 10; DPP6-KO = 24 ± 2 cm/s, *n* = 10, Student’s *t*-test, *t*_(18)_ = 1.73, *p* > 0.05), suggesting that the knockouts show comparable motor activity to WT mice. During the probe trial test in which the platform was removed, DPP6-KO mice were significantly slower to reach the platform quadrant (Figures 1D,E, WT = 11 ± 2 s, DPP6-KO = 17 ± 2 s, Student’s *t*-test, *t*_(18)_ = 2.15, *p* < 0.05) and exhibited less platform crossing (Figure 1F, WT = 6 ± 0.6 entries, DPP6-KO = 4 ± 0.8 entries, Student’s *t*-test, *t*_(18)_ = 2.18, *p* < 0.05) compared to WT mice indicating impaired spatial memory in DPP6-KO mice compared to WT mice.

### T-Maze

**Figure 2:**
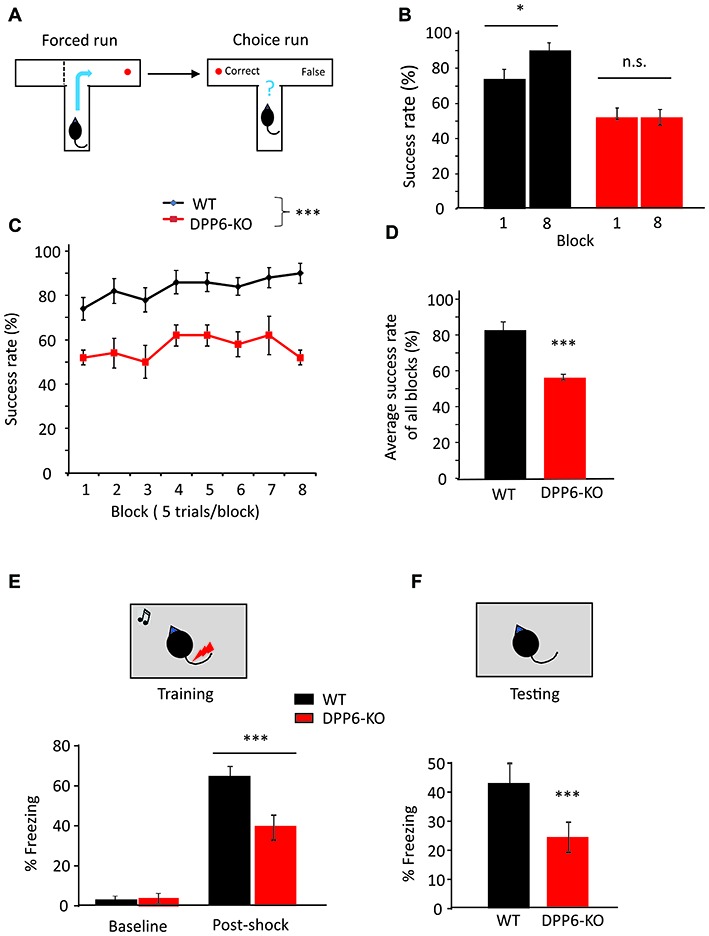
DPP6-KO mice show impaired spatial working memory in the T-maze and contextual fear conditioning tasks. **(A)** Schematic showing the experimental design in forced run and choice run of the T-maze task. **(B)** Percentage of correct choices for mice during the learning phase. **(C)** Percentage of correct choices comparing Day 1 to Day 8. **(D)** Percentage of correct choices for average scores over 8 days (*n* = 10 each group), compared with Student’s *t*-test. **(E,F)** Contextual fear conditioning task, at 24 h after training, DPP6-KO mice show a significant reduction in time exhibiting freezing behavior when placed back in the test chamber (*n* = 10 each group) (n.s. = not significant, *p < 0.05, ***p < 0.001).

We next used the rewarded T-maze alternation task to detect hippocampus-dependent memory and spatial learning dysfunction in DPP6-KO mice (Chapman et al., 1999). During the first trial (forced; Figure 2A, left), one arm of the maze was blocked and the mouse entered the opposing arm where it received a reward. On the second trial (choice; Figure 2A, right), the mouse was rewarded only if it chose the unvisited arm. We found that the success rate of WT mice increased over the course of the training in the task for 40 trials during eight consecutive days (Figure 2B, main effect of genotype, one-way ANOVA (*F*_(1,18)_ = 64.48, *p* < 0.001), no effect of either session or Genotype × session interaction). When comparing Day 1 to Day 8 over the 8-block training, however, DPP6-KO mice showed no significant learning (Figure 2C, WT = 74% to 90%, *p* < 0.05; DPP6-KO = 52% to 52%, *p* > 0.05). After training, WT but not DPP6-KO mice showed a significantly increased success rate (Figure 2D, WT = 83 ± 5%, DPP6-KO = 56 ± 2%; Student’s *t*-test, *t*_(18)_ = 10.36, *p* < 0.001). There was no significant difference in the latency to enter either the choice or forced run throughout the training period (data not shown), suggesting that the spatial working memory impairment seen in DPP6-KO mice was not due to hyperactivity during the task.

### Fear Conditioning

Next, we examined the WT and DPP6-KO mice in the contextual fear conditioning task. In the training session, mice were placed in a conditioning chamber where they were given three pairings of tone and foot shocks; the animal learns to fear the tone. Twenty-four hours after the training, we performed context testing, where the mice were exposed to the same conditioning chamber and their freezing behavior was assessed. The results indicate that DPP6-KO mice show decreased freezing immediately after the tone/shock pairing training compared to WT (Figure 2E, main effect of genotype, ANOVA (*F*_(1,18)_ = 8.84, *p* < 0.01), main effect of training, ANOVA (*F*_(1,18)_ = 179.46, *p* < 0.01); genotype × training interaction, ANOVA (*F*_(1,18)_ = 14.72, *p* < 0.01)). During the context retrieval, DPP6-KO mice showed significantly decreased freezing response and fear memory compared to WT (~40% lower, Figure 2F, *p* < 0.01). To examine sensory processing in DPP6-KO mice, we performed the Hot Plate test (WT and DPP6-KO mice both *n* = 10 mice/group and ~4 m old). We recorded the time when the mouse starts licking the hind paw on a 55°C hot plate. We found no significant difference between WT and DPP6-KO mice (WT = 14.7 ± 0.5 s, DPP6-KO = 13.7 ± 0.4 s, Student’s *t*-test, *t*_(18)_ = 1.54, *p* = 0.14). This finding further demonstrates that learning and memory are impaired in DPP6-KO mice.

### Spatial Object Recognition and Novel Object Recognition

Object recognition memory is another type of declarative memory that critically depends on hippocampal function. We performed two object recognition behavioral tasks to measure spatial and non-spatial memory.

**Figure 3:**
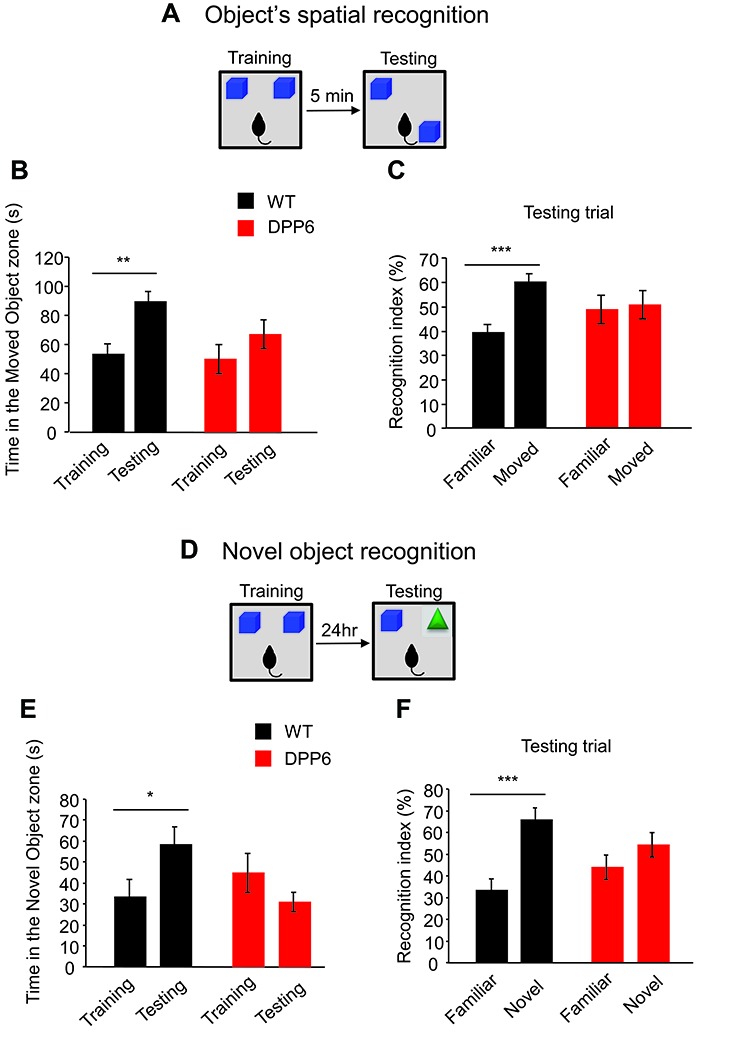
DPP6-KO mice show impaired recognition memory. **(A)** Schematic of the spatial object recognition paradigm. **(B)** WT mice show increased time spent in exploring the moved object, while DPP6-KO mice do not show a difference in time exploring the moved object at test trial compared to training trial. **(C)** DPP6-KO mice showed no recognition between familiar and moved object but WT showed significant increase of recognition of the moved object. **(D)** Schematic of the Novel object recognition paradigm. **(E)** WT mice spend more time exploring the novel object at testing trial. However, DPP6-KO mice do not show a preference for the novel object over a familiar object. **(F)** WT mice showed greater recognition of the novel object, but DPP6-KO mice show no significant preference between the novel and familiar object (*p < 0.05, **p < 0.01, ***p < 0.001).

During spatial object recognition experiments (Figures 3A–C), two identical familiar objects were placed in the arena and the animal could explore freely for 10 min. After a delay of 5 min, one of those objects was moved to a different location and the animal could explore once more for 10 min. Animals with normal hippocampal function will prefer to explore the object that has been moved rather than the one that is left in place. In spatial object recognition tasks, both WT and DPP6-KO mice spent a similar amount of time with the two objects in an open-field box during the training trial (WT^Training object-1^ = 58 ± 8 s, WT^Training object-2^ = 54 ± 7 s, *p* > 0.05; DPP6-KO^Training object-1^ = 50 ± 8 s, DPP6-KO^Training object-2^ = 50 ± 10 s, *p* > 0.05). In the probe trial in which one of the objects was relocated to a novel position in space, WT mice exhibited a strong preference and spent more time with the moved object in the novel location compared to the training trial (Figure 3B, *t*_(18)_ = 3.73, *p* < 0.01). WT mice also showed a significantly higher recognition index for the moved object than the static object in the testing trial (Figure 3C, *t*_(18)_ = 4.99, *p* < 0.001). However, DPP6-KO mice showed no significant difference in exploration of the moved object (Figure 3B, *t*_(18)_ = 1.23, *p* > 0.05), and the recognition index shows no significant increase between the moved and static object (Figure 3C, *t*_(18)_ = 0.03, *p* > 0.05). These results suggest that DPP6-KO mice have impaired memory in the spatial object recognition task.

The novel object recognition task tests the ability to remember the object itself (Figures 3D–F). In the probe trial of the novel object recognition experiment 24 h after training, a familiar object was replaced by a novel object. Compared to DPP6-KO, WT mice showed an increased preference for the novel object (Figure 3E, WT, *t*_(18)_ = 2.18, *p* < 0.05; DPP6-KO, *t*_(18)_ = 1.35, *p* > 0.05). WT mice also showed a significantly higher recognition index in the novel object than the familiar object in the testing trial (Figure 3F, WT, *t*_(18)_ = 4.48, *p* < 0.001; DPP6-KO, *t*_(18)_ = 1.49, *p* > 0.05). Taken together, these data demonstrate that DPP6-KO mice have impaired hippocampus-dependent recognition memory.

### Open Field and Home-Cage Locomotor Activity

**Figure 4:**
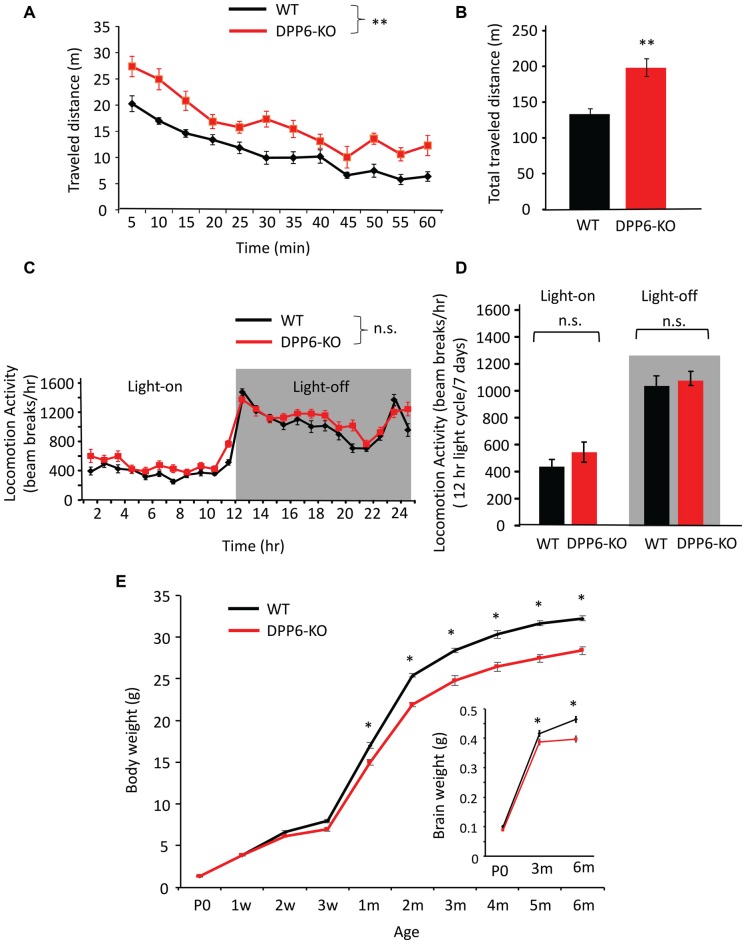
DPP6-KO mice locomotor activity in the open field and home cage. **(A)** In the open field task, DPP6-KO mice show increased locomotor activity in a novel environment for 60 min. compared to WT. **(B)** DPP6-KO mice show increased total distance traveled in the open field test compared to WT mice. **(C)** DPP6-KO mice show normal locomotion in a familiar environment, as measured by 24-h continuous monitoring of mouse movements in a home cage-like environment. **(D)** The distance moved during light-on or light-off phase. **(E)** The body weight of DPP6-KO mice is lower from 1 month after birth and remains smaller throughout their adult life to 6 months compared to WT at the same ages (*n* > 20 each group). Inset: DPP6-KO mice also show smaller brains at P0, 3 and 6 months compared to WT (n.s. = not significant, *p < 0.05, **p < 0.01).

As locomotor activity can affect the interpretation of cognitive related tasks we set out to screen locomotor activity levels in both a novel (open field) and familiar (home cage) environment. Open field activity measures are used to assess locomotive and behavioral activity levels in a novel environment. In the open field test, DPP6-KO mice showed increased locomotion compared with their WT controls (Figures 4A,B, main effect of genotype, ANOVA (*F*_(1,17)_ = 21.62, *p* < 0.01), effect of time (*F*_(11,182)_ = 39.30, *p* < 0.01), no effect of genotype × time interaction) and total traveled distance was greatly increased compared to WT (Figure 4B, WT = 138 ± 7 min, DPP6-KO = 198 ± 12 min, Student’s *t*-test, *t*_(17)_ = 3.01, *p* < 0.01). We also analyzed the movement and rest time. DPP6-KO mice show significantly more movement time (WT = 35 ± 11 min, DPP6-KO = 40 ± 13 min; Student’s *t*-test, *t*_(17)_ = 2.14, *p* < 0.05) and less resting time during the task compared to WT (WT = 25 ± 8 min, KO = 20 ± 6 min; Student’s *t*-test, *t*_(17)_ = 2.14, *p* < 0.05). There was no significant difference between DPP6-KO mice and WT in the time spent in the center (data not shown). We also tested anxiety level in DPP6-KO mice by the elevated plus-maze. DPP6-KO mice displayed decreased anxiety-like behavior with increased time spent in the open arms (WT = 62 ± 4 (s), *n* = 12; DPP6-KO = 109 ± 19 (s), *n* = 11, Student’s *t*-test, *t*_(21)_ = 2.08, *p* < 0.05. We also performed locomotor activity monitoring in the home cage by 24 h continuous monitoring for 6 days (Figures 4C,D). In the home cage, locomotor activity was not significantly different between WT and DPP6-KO mice either in light-on or light-off times (Figure 4C, no effect of genotype, ANOVA, *p* > 0.05; no effect of genotype × time interaction, *p* > 0.05) or light-on/off (Figure 4D, no effect of genotype, ANOVA, *p* > 0.05; no effect of Genotype × Time interaction, *p* > 0.05). When pooled, light-on and light-off times were not significantly different between WT and KO groups (data not shown). These results show that DPP6-KO mice have normal locomotion in the home cage environment, suggesting that their increased locomotor activity in the open field could be due to enhanced anxiety levels in the novel environment.

### DPP6 Loss Leads to Lower Body and Brain Weight

Low birth weight is associated with neurodevelopmental disabilities in humans, and DPP6 gene loss is related to developmental disorders (Egger et al., 2014; Bock et al., 2016). To better understand the DPP6-KO mouse phenotype, we measured the body weight from P0 to 6 months of age of WT and DPP6-KO mice at 10 different age points (Figure 4E). We found that DPP6-KO mice have significantly lower body weight (8%–15% less) compared to WT (Figure 4E, *n* = 20–30 mice per age, *p* < 0.0001, *F*_(9,417)_ = 15.33, Two-way ANOVA). We confirmed that reduced body weight of DPP6-KO mice occurred despite normal feeding behavior compared to WT adult mice (data not shown). We also measured the brain weight of P0, 3-month and 6-month old mice. We found that the brain weight of DPP6-KO mice was significantly lower than WT at 3 and 6 months (Figure 4E, insert, *n* = 15–28 per age, *p* = 0.0001, *F*_(2,104)_ = 9.866, Two-way ANOVA). Studies indicate that the DPP6 gene is associated with microcephaly and mental retardation (Liao et al., 2013; Lucchese and Kanduc, 2017).

### DPP6 Loss Leads to Developmental Deficits in Synaptic Formation and Stabilization

**Figure 5:**
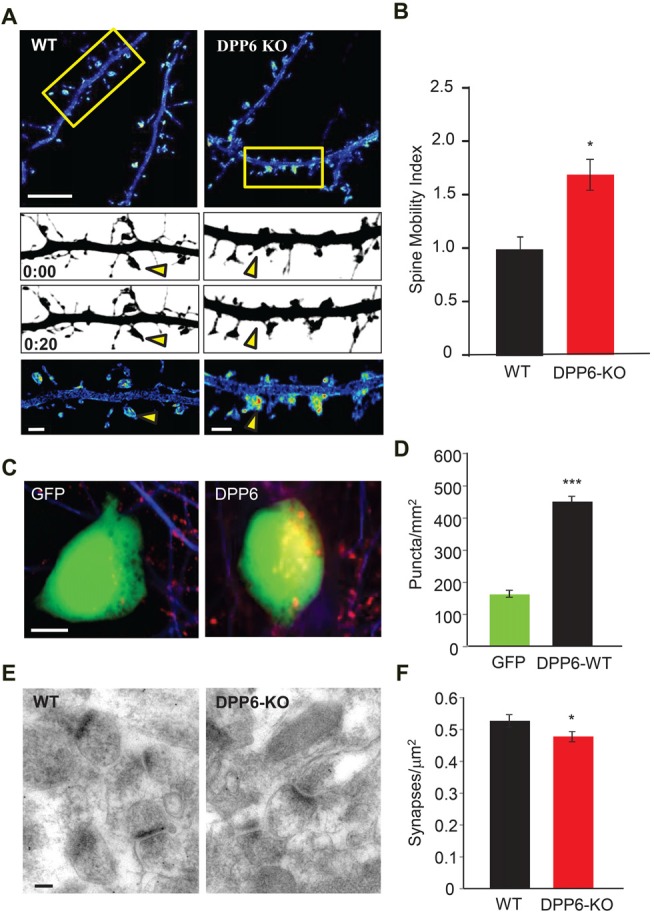
DPP6 regulates synapse formation and stabilization. **(A)** Pseudocolored, summed intensity-projection photomicrographs of cultured hippocampal mouse neurons expressing mCherry. Warmer colors represent regions of higher motility. The boxed areas in the top images are shown in greater magnification in the bottom three panels. Gray-scale images show spine morphology at the beginning (above; 0:00) and end (below; 0:20) of the imaging session. Yellow arrows depict regions of differing motility between WT and DPP6-KO neurons. Scale bars: (top) 10 μm; (bottom) 2 μm. **(B)** Graphs represent the mean spine motility index ± SEM in either WT or DPP6-KO neurons (*n* = 10–11). **(C,D)** HEK293T cells expressing DPP6-GFP or control GFP only, HEK293T cells (labeled with GFP) were co-cultured with hippocampal neurons for 48 h and stained for synapsin. The synapsin puncta that formed on HEK293T cells expressing DPP6 are significantly denser and larger than those formed on control HEK293T cells. *n* = 46 for control, *n* = 77 for DPP6. Scale bars represent 10 μm. **(E,F)** Synapse density in the CA1 stratum radiatum in WT and DPP6-KO mice. Mean synapse density is reduced in DPP6-KO mice relative to WT. Two mice for each genotype; Scale bar is 100 nm (*p < 0.05, **p < 0.01, ***p < 0.001).

In our previous study, we reported that DPP6 plays a novel role in dendritic filopodia formation and stability, subsequently impacting spine and synapse number and interactions with the extracellular matrix (Lin et al., 2013). Dendritic spines are highly dynamic and regulate synaptic efficacy; they are heterogeneous and dynamic structures that receive and transmit excitatory synaptic input to regulate neuronal excitability and circuit function. Numerous human brain disorders are associated with altered dendritic spine structure including autism, and autism-like disorders including Angelman, Rett and Fragile X syndromes (Penzes et al., 2011). Because DPP6 regulates synaptic development and function (Lin et al., 2013), we sought to measure dendritic spine dynamics in WT or DPP6-KO mouse cultured neurons. To monitor spine motility, we transfected cultured hippocampal neurons with mCherry fluorescent protein as a fiducial marker of cell morphology. Dendritic segments were imaged at 1 min intervals and the cumulative motion of dendritic spines was measured over a 20-min period. When compared to WT, DPP6-KO mouse neurons had higher motility based on a normalized spine motility index (Figures 5A,B. DPP6-KO (1.458 ± 0.157), Student’s *t*-test, *t*_(19)_ = 2.59, *P* < 0.05), supporting a role for DPP6 as a synaptic adhesion molecule. This result suggests that the destabilization of spines in DPP6-KO mice might affect synapse formation. To examine this, we performed a mixed-culture assay (Figures 5C,D) to determine if DPP6 promotes synaptogenesis. To test this possibility, we expressed either GFP or DPP6-GFP in HEK293T cells for 48 h and seeded the cells onto maturing hippocampal neurons (DIV10). The number of synapsin puncta formed on HEK293T cells expressing DPP6-GFP was significantly increased relative to those expressing GFP alone (Figures 5C,D, GFP, *n* = 46; DPP6, *n* = 77; *t*_(121)_ = 3.37, *p* < 0.001), indicating that DPP6 is a synaptogenic protein. Additionally, in the hippocampal CA1 region in adult mice, we measured total synapse number with post-synaptic density (PSD) as a standard from each section imaged by electron microscopy; we found that the number of synapses in WT is greater than in KO (Figures 5E,F, Table 1. WT, *n* = 174 images; DPP6-KO, *n* = 194 images, *p* < 0.001). This result is consistent with our previous finding of decreased spine density and mEPSC frequency in DPP6-KO neurons (Lin et al., 2013). Taken together, our results suggest that DPP6 plays an important role in synapse formation and stabilization.

**Table 1:** Synaptic structures of the hippocampal CA1 region in WT and DPP6 knockout (DPP6-KO) adult mice.

	WT (mean ± SEM)	DPP6-KO (mean ± SEM)	*T* value (degree of freedom)
PSD surface area (nm^2^)	10611 ± 298	7157 ± 161***	*T*_(448)_ = 10.7
	*n* = 203	*n* = 247
PSD perimeter (nm)	524 ± 10	431 ± 6***	*T*_(448)_ = 8.2
	*n* = 203	*n* = 247
PSD thickness (nm)	54 ± 0.7	44 ± 0.5***	*T*_(525)_ = 11.7
	*n* = 238	*n* = 289
PSD length (nm)	244 ± 4	205 ± 3***	*T*_(435)_ = 7.3
	*n* = 220	*n* = 217
PSD mean intensity	77 ± 0.8	65 ± 0.9***	*T*_(448)_ = 9.8
	*n* = 203	*n* = 247
Cleft width (nm)	16.8 ± 0.3	17.5 ± 0.3^n.s^	*T*_(387)_ = 1.7
	*n* = 176	*n* = 213
Cleft mean intensity	94 ± 1	81 ± 1***	*T*_(387)_ = 7.8
	*n* = 176	*n* = 213
Spine head area (nm^2^)	94330 ± 3592	66264 ± 2204***	*T*_(509)_ = 6.8
	*n* = 241	*n* = 270
Spine perimeter (nm)	1163 ± 22	994 ± 16***	*T*_(509)_ = 6.4
	*n* = 241	*n* = 270
Spine mean intensity	127 ± 0.9	118 ± 1***	*T*_(509)_ = 6.4
	*n* = 241	*n* = 270
Synapses density (Synapses/μm^2^)	0.53 ± 0.02	0.48 ± 0.01*	*T*_(366)_ = 2.0
	*n* = 174 images	*n* = 194 images

*p < 0.05; ***p < 0.001; n.s. = not significant, Student’s t-test.

### DPP6 Loss Leads to Synaptic Structure Deficits in the Hippocampal CA1 Region of Adult Mice

**Figure 6:**
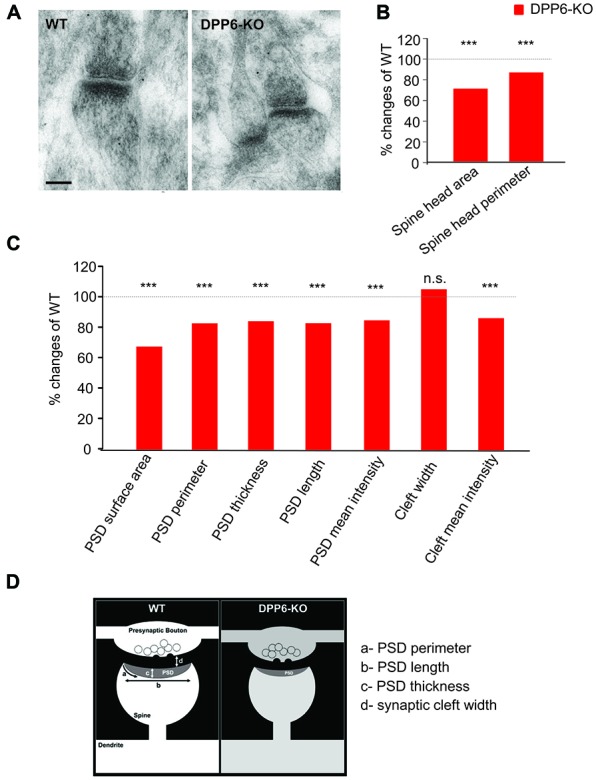
DPP6 mice have developmental deficits in synaptic structures of the hippocampal CA1 Region. **(A)** Representative images of electron micrographs showing the synaptic contacts with presynaptic vesicles, and postsynaptic densities in dendritic spines in the CA1 region of the WT (left) and DPP6-KO (right) hippocampus. Scale bar is 100 nm. **(B)** Measurement of dendritic spine head size. The spine head area and perimeter are reduced 30% and 15% in DPP6-KO mice relative to WT as 100%. Student’s *t*-test, *p* < 0.001. **(C)** Summary of postsynaptic density measurements. **(D)** Schematic drawing showing a synaptic contact with presynaptic vesicles and a postsynaptic density in a dendritic spine. Approximate measured relative dimensions are displayed to compare DPP6-KO with WT (n.s. = not significant, ***p < 0.001).

In the hippocampus, dendritic spines vary greatly in their dimensions, even along a single dendritic segment. The size of a dendritic spine correlates with the number of presynaptic neurotransmitter vesicles and scales with synaptic strength (Segal, 2005). We have reported that the novel DPP6 developmental effects are functionally relevant into adulthood. Here, we used electron microscopy to determine whether DPP6 affects synaptic structures in the hippocampal CA1 region in adult mice. First, we measured the size of the spine head, including surface area, perimeter and mean intensity of the area from each section imaged (Figures 6A,B). The results presented in Table 1 showed that WT spines have significantly larger spine head area, longer perimeter and stronger mean intensity compare to KO mice, which showed a 30% decrease in spine head area and a 15% smaller perimeter (Figure 6B). The PSD is an electron dense structure that contains receptors, scaffolding proteins, and signaling complexes important for synaptic transmission and plasticity. To get more detail on synaptic organization in DPP6-KO mice, we measured the PSD size, which included its surface area, perimeter, thickness, length, and mean intensity. The PSD size of spines in the DPP6-KO hippocampal CA1 region was significantly decreased relative to WT mouse (32% smaller surface area, 18% shorter perimeter, 16% less thickness and 18% less length; also, the mean intensity of PSD was 15% weaker than in WT; Figure 6C, Table 1). Because we had previously established a role for DPP6 in interactions with the extracellular matrix and in regulation of spine stability, we also measured the width and mean intensity of the synaptic cleft. Our results showed that the width of synaptic cleft is not significantly different between WT and DPP6-KO mice (Figure 6C, Table 1), but the mean intensity of synaptic cleft is significantly decreased by about 15% in DPP6-KO compared to WT (Figure 6C, Table 1).

## Discussion

Dendritic spines are postsynaptic chemical and electrical compartments that are widely considered to be important for synaptic plasticity. Changes in spine structure during synaptic plasticity are related to changes in synaptic efficacy, learning and memory and other cognitive processes. We have previously shown (Sun et al., 2011; Lin et al., 2013) that DPP6-KO mice have fewer functional spines. Here, we extend these findings with live-imaging and EM results which indicate that DPP6 plays important roles in glutamatergic synapse structure, formation and motility. Moreover, DPP6 deletion leads to behavioral impairments in recognition and spatial learning and memory. Differences in spine motility and size in DPP6-KO mice suggest that DPP6 regulates the development of synaptic structures that are important for the acquisition and retention of learned behaviors.

### DPP6 Acts as a Synaptic Cell Adhesion Protein

Synapse formation, maintenance and plasticity rely on coordination between multiple types of structural proteins spanning the synaptic cleft. Our previous work identified a novel role for DPP6 in the regulation of dendritic filopodia formation and stability indicating that DPP6 plays roles in synaptic cell adhesion. DPP6 also interacts with the extracellular matrix protein fibronectin to impact hippocampal synaptic development and function. In this study, we extend these findings to show that DPP6 regulates glutamatergic synapse formation and regulates spine stabilization using time-lapse imaging of cultured neurons and in adult hippocampal CA1 EM images (Figures 5, 6). These data indicate that there are fewer glutamatergic synapses formed in the DPP6-KO mouse hippocampal CA1 region, where we found spines have smaller spine head and PSD areas. In addition, we found that the mean intensity in the synaptic cleft in DPP6-KO mice is significantly decreased compared to WT, suggesting that there are fewer proteins in the cleft of the DPP6-KO. Perhaps the loss of DPP6 impairs synapse stability and leads to disorganization of trans-synaptic cell adhesion molecules bridging the synaptic cleft.

We found that DPP6-KO has reduced electron density in the PSD region, which contains receptors, channels, scaffolding proteins and signaling complexes. This lower density could indicate a loss of some PSD proteins, including DPP6 and its binding partners, such as Kv4.2, and perhaps other proteins indirectly associated with these complexes. Thus, DPP6 loss may not only affect cell adhesion, but also intercellular trans-synaptic signaling. These findings will be extended in future studies aimed at identifying DPP6 binding partners that co-regulate synapse formation and function. Additional work is also required to determine if and how DPP6 affects GABAergic synapses, which were not a topic of the current study.

### DPP6 Affects Synaptic Development and Plasticity

The present imaging and EM results discussed above, together with our previous work (Lin et al., 2013), demonstrate a clear role for DPP6 in the development of dendritic filopodia, spines and glutamatergic synaptic currents. We showed previously that, compared with WT controls, CA1 pyramidal cells from adult DPP6-KO mice exhibited a significant reduction in spine density in both apical and basal dendritic location and a sparser dendritic arbor. The loss of spines leads to functional impairments including a reduction in miniature excitatory postsynaptic current frequency, but not amplitude, suggesting that DPP6-KO mice have fewer functional excitatory glutamatergic synapses in CA1 neuron dendrites. The effects of DPP6 loss were not replicated in neurons lacking Kv4.2 suggesting that DPP6 has synaptic developmental functions independent of its role as a Kv4 auxiliary subunit. However, in accordance with this role, DPP6-KO mice also display reduced dendritic A-type K^+^ currents, resulting in hyperexcitable dendrites with a decreased threshold for calcium-spiking. These changes lead to alterations in synaptic plasticity in the hippocampus, thought to underlie learning and memory (Sun et al., 2011). It will take additional efforts to distinguish which and how these various cellular consequences of DPP6 loss contribute to the present findings of poor memory performance. DPP6 mutations have been associated with ASD (Marshall et al., 2008; Noor et al., 2010; Egger et al., 2014; Bock et al., 2016; Maussion et al., 2017) and other neuropsychiatric pathologies (Marshall et al., 2008; Noor et al., 2010; Egger et al., 2014; Bock et al., 2016; Maussion et al., 2017; Noor and Zahid, 2017) and intellectual difficulties (Penzes et al., 2011; Egger et al., 2014; Bock et al., 2016; Lucchese and Kanduc, 2017). Identifying the precise mechanism underlying learning and memory deficits in DPP6 mice may help in developing therapeutic targets for the learning deficits that occur in individuals with neurodevelopmental disorders.

In addition to the synaptic and behavioral differences reported here, we found DPP6 mice to be significantly smaller than WT in body and brain weight throughout postnatal development and into adulthood. That the brain weight was significantly lower for DPP6-KO at P0, when there is not yet any significant difference in body weight compared to WT, suggests that the brains of DPP6-KO mice have an embryonic developmental delay. Normal body weight at P0 but decreasing body weight as the DPP6 mice age suggests a metabolic phenotype developing after birth. In fact, a metabolic link between DPP6 and the glucose-insulin pathway has been previously reported (Imai et al., 2008). Future studies may indicate a functional role for Kv4 K^+^ channels in glucose-stimulated insulin secretion which may be modified by DPP6 expression.

### DPP6 Loss Affects Learning and Memory

Previous results suggest a potential effect of DPP6 on intellectual function and memory formation (Lin et al., 2013; Uddin and Singh, 2017). Accordingly, in the present study, we found that DPP6-KO mice show clear deficits in spatial learning/memory phase of the Morris water maze, T-maze, and object recognition memory (Figures 1–3). In addition, DPP6-KO mice displayed impaired contextual learning in the fear conditioning test.

The hippocampus plays an important role in learning and memory (Eichenbaum et al., 1992; Jarrard, 1993). Rodents with hippocampal lesions exhibit robust delay-dependent deficits in the Morris water maze (Morris et al., 1982), T-maze task (Hock and Bunsey, 1998) as well as contextual (place-based) fear conditioning (Kim and Fanselow, 1992), which has led to the conclusion that the rodent hippocampus is primarily involved in spatial learning and memory (Eichenbaum et al., 1990). Lesions in the dorsal CA1 specifically can also produce deficits in the Morris Water Maze (Stubley-Weatherly et al., 1996) as well as in the T-maze (Hernández-Rabaza et al., 2007). Because DPP6 is expressed in, and in the current study deleted from, the entire hippocampus, we cannot conclude that the behavioral changes observed here reflect the changes observed in dorsal CA1 neurons. They could alternatively reflect the deletion, and perhaps similar synaptic changes, elsewhere in the hippocampus.

Both learning and contextual conditioned fear were impaired in DPP6-KO mice, consistent with a role for DPP6 role in memory. However, this result could be influenced by hyperactivity seen in the DPP6-KO mice when exposed to novel environments. Lesions of the hippocampus have long been known to induce locomotor hyperactivity in novel environments (Jarrard, 1968; Bannerman et al., 2012), though they also reportedly increased locomotor activity in the home cage (Jarrard, 1968), which was not observed in the current study. This hippocampal impairment-induced hyperactivity can disrupt freezing behavior, affecting performance in fear conditioning tests even when memory is intact (McNish et al., 1997), making it difficult to assess memory in this classic test. More recent studies using lesion and optogenetic silencing of neurons have localized both spatial memory impairment and hyperlocomotion effects of hippocampal dysfunction to the dorsal hippocampus, and, in particular, dorsal dentate gyrus (Anagnostaras et al., 1999; Kheirbek et al., 2013). Locomotor hyperactivity clearly affects exploratory tasks, such as the open field and elevated plus maze (Kheirbek et al., 2013), as well as tests in which exploration competes with freezing, but it is also likely to influence other behaviors such as the object recognition, water maze and T-maze tasks to varying degrees. Hyperactivity can be difficult to control for, but DPP6-KO mice showed normal swim speed in the water maze test and normal latency in both forced and choice run of the T-maze test, like WT controls, suggesting that novelty induced hyperactivity did not strongly influence the results seen in these studies. Object location memory is hippocampal dependent, and dependent on CA1 specifically (Gilbert and Kesner, 2004; Hunsaker et al., 2007), consistent with DPP6-KO impairment in CA1-dependent spatial memory. Memory for objects themselves (novel object recognition) is often described as not requiring the hippocampus, but a long delay, like that used in the current study, disruption of CA1 does impair object recognition (Hammond et al., 2004), suggesting that effects of DPP6-KO in this test is consistent with CA1 specific synaptic impairment as well. The consequences of DPP6-dependent behavioral effects attributable to other brain regions may be explored in future research.

## Author Contributions

LL, JGM and R-MK designed the experiments, performed experiments, analyzed the data and wrote the manuscript. RSP designed the experiments and analyzed the data. JJG and Y-XW performed experiments and analyzed the data. DA performed experiments. HAC and DAH designed the experiments and wrote the manuscript.

## Conflict of Interest Statement

The authors declare that the research was conducted in the absence of any commercial or financial relationships that could be construed as a potential conflict of interest.
